# Inhomogeneous Oxygen Vacancy Distribution in Semiconductor Gas Sensors: Formation, Migration and Determination on Gas Sensing Characteristics

**DOI:** 10.3390/s17081852

**Published:** 2017-08-10

**Authors:** Jianqiao Liu, Yinglin Gao, Xu Wu, Guohua Jin, Zhaoxia Zhai, Huan Liu

**Affiliations:** 1 College of Information Science and Technology, Dalian Maritime University, Linghai Road 1, Ganjingzi District, Dalian 116026, China; jingh@dlmu.edu.cn (G.J.); shirlyllei@dlmu.edu.cn (Z.Z.); 2 College of Marine Electrical Engineering, Dalian Maritime University, Linghai Road 1, Ganjingzi District, Dalian 116026, China; yinglin_lynn1997@126.com (Y.G.); ddwx_1996@126.com (X.W.); 3 School of Optical and Electronic Information, Huazhong University of Science and Technology, 1037 Luoyu Road, Wuhan 430074, China

**Keywords:** semiconductor gas sensor, oxygen vacancy, inhomogeneous distribution, sensing mechanism, defect behavior, kinetics

## Abstract

The density of oxygen vacancies in semiconductor gas sensors was often assumed to be identical throughout the grain in the numerical discussion of the gas-sensing mechanism of the devices. In contrast, the actual devices had grains with inhomogeneous distribution of oxygen vacancy under non-ideal conditions. This conflict between reality and discussion drove us to study the formation and migration of the oxygen defects in semiconductor grains. A model of the gradient-distributed oxygen vacancy was proposed based on the effects of cooling rate and re-annealing on semiconductive thin films. The model established the diffusion equations of oxygen vacancy according to the defect kinetics of diffusion and exclusion. We described that the steady-state and transient-state oxygen vacancy distributions, which were used to calculate the gas-sensing characteristics of the sensor resistance and response to reducing gases under two different conditions. The gradient-distributed oxygen vacancy model had the applications in simulating the sensor performances, such as the power law, the grain size effect and the effect of depletion layer width.

## 1. Introduction

It has been more than half of a century since the first invention of semiconductor gas sensors was made from two pioneer research groups led by Seiyama and Taguchi in 1962 [1,2]. This type of sensor soon found its wide applications in gas leakage alarms [3], environment monitoring [4] as well as the mine and oil industry [5]. Many researchers were attracted by the importance of semiconductor gas sensors in the past decades. Considerable effort has been invested in the new generations of sensors, including semiconductor bulks [6], thick films [7,8,9,10,11,12,13,14], thin films [15,16,17,18,19,20,21,22,23,24,25,26] and nanostructured composites [27,28,29,30,31,32,33,34,35], which were made of different materials. These sensors hold the advantages of compatibility with microelectronic technology, low cost, being free of toxicity and good stability. However, most of the semiconductor gas sensors require high operating temperatures, which lead to high power dissipation and risks of explosion [36,37,38]. Thus, some researchers endeavored to develop the gas sensors that could work at low operating temperatures [39,40,41], even at room temperature [42,43,44,45,46], in order to meet the requirements of the novel electronic devices. Recently, gas sensors involving semiconductive quantum dots were successfully developed, which have great potential for gas detection at room temperature. Therefore, the application of quantum dots in gas detection has become a new hot topic in the frontier investigations of semiconductor gas sensors [47,48,49,50,51,52,53,54,55,56,57,58]. At the same time, some researchers started to seek theoretical understandings of semiconductor gas sensors, with the appearance of a variation in resistivity during gas detection. For a typical semiconductor gas sensor, it had a core-sensing body that contained millions of tiny semiconductive grains. The sensing mechanism was considered to comprise three parts, which are namely the receptor function, the transducer function and the utility factor [59]. The receptor function describes how a single grain responds to oxygen in the aerial atmosphere and to stimulant gases [60,61,62,63]. Several valuable conclusions have been made in the previous reports related to the receptor function. The sintered semiconductor grains are nonstoichiometric, such as SnO_2−X_, and there are inherent oxygen vacancies (V_O_) in the system [64]. The defects on the grain surface provide adsorption sites for aerial oxygen. The types of adsorbed oxygen are found to be O^−^, O_2_^−^ and O^2^^−^, which are dependent on the operating temperature [65,66,67,68]. They also determine the sensor sensitivity, which is usually known as the power law exponent for semiconductor gas sensors. The adsorbed oxygen can seize quasi-free electrons near the surface of grains and produce a depletion layer [69]. It was concluded that most of adsorption sites are left vacant due to the shortage of electron supply, meaning the amount of adsorbed oxygen is controlled by the number of electrons in the depletion layer [70]. When the grain is exposed to reducing gases, the adsorbed oxygen is consumed by gas molecules and the seized electrons are released back to the depletion layer, resulting in a grain response of decreased resistivity. If an oxidizing gas is introduced, the gas molecules competitively adsorb on the grain surface with aerial oxygen, leaving a grain response of increased resistivity. The transducer function indicates how the response of each grain is transduced into device resistance. In a semiconductor system of grain matrix, the double Schottky barriers for electrons are established at the grain boundaries, where the grains interact with each other. The height of the Schottky barrier determines the device resistance, which therefore changes in the gas detection. In another angle of view, the tunneling effect may take place at various circumstances [71]. Some researchers believe that it is the better one to interpret the transducer function [72]. The third one, the utility factor, describes how the device response is attenuated in an actual sensor due to the gas consumption when the stimulant gas diffuses into the sensing body. The gas diffusion theory [62,73,74] was proposed to formulate the utility factor of semiconductor thin film gas sensors in 2001 and after this time, some amendments have been made to extend the application of the theory [75,76]. Thus, the utility factor became the best understood one among the three parts.

The knowledge above establishes the framework of the fundamental sensing mechanism of a semiconductor gas sensor. However, there are still some essential questions that are not answered satisfactorily. Among these questions, the effects and behaviors of oxygen vacancies have received more and more attentions from researchers. The oxygen vacancy was found to be the predominant defect, which occurred independently of the oxygen partial pressure. When the oxygen partial pressure was lowered, the oxygen vacancy formation enthalpy decreased and became exothermic under very O-poor condition [77]. Oxygen vacancies at the surface facilitated oxygen adsorption and the gas response was remarkably enhanced by the defects [78]. They created a special chemistry state from the grain surface process by improving the adsorption and enhancing the charge transfer from the surface to the adsorbates [79]. The gas-sensing properties of the ZnO nanowalls for NO_2_ detection benefited from modification of oxygen vacancies [80,81,82]. H_2_O_2_ pretreatment and annealing were employed to generate oxygen vacancies on ZnO surface in order to enhance the gas-sensing response [83]. The first principle calculation demonstrated that the bandgap was narrowed when the oxygen vacancy was increased in the ZnO sensors [84]. The defects may also decrease the lattice constant in ZnO crystals [85]. The existence of oxygen vacancies benefited the NO_2_ adsorption on nanowire surface and markedly increased the interaction between the molecule and vacancy-defected surface. Three types of stable oxygen vacancies increased the number of transferred electrons to being 4–6 times more than that in the stoichiometric nanowire, which indicated a positive effect of oxygen vacancy on NO_2_-sensing response [86]. However, a contrary research from density functional theory (DFT) calculations proposed that the oxygen vacancy exerted negative effects on the sensing ability of WO_3_ materials [87]. The DFT study also concluded that V_O_ migrations were most likely to occur under low oxygen-defect concentration [88].

In the theoretical investigation of sensing mechanism, the density distribution of V_O_ is considered to be uniform for convenience in mathematics [89]. However, it is unlikely that oxygen defects distribute homogeneously in an actual grain. The effect of V_O_ distribution is not taken into consideration in the sensing theory of semiconductor gas sensors. Therefore, a series of investigations concerning inhomogeneous V_O_ distribution have been carried out [90,91,92,93,94,95,96]. On the basis of the influence of cooling and annealing process on the sensor properties, the V_O_ dynamics of diffusion and exclusion were discussed, which was used to create a gradient profile of defects. The model of the gradient-distributed oxygen vacancies (GDOV model) was proposed for formulating the V_O_ density and calculating its determination on gas-sensing characteristics, such as resistance and sensor response to stimulant gases. In the present work, the main conclusions of the model are reviewed from the derivation to the validity of simulated sensor characteristics. Furthermore, the application of the model is extended to several cases, such as simulating the power law, the grain size effect and the effect of depletion layer width. The successes and reservations of the GDOV model are also discussed.

## 2. Gradient-Distributed Oxygen Vacancy Model

### 2.1. Model Description

**Figure 1:**
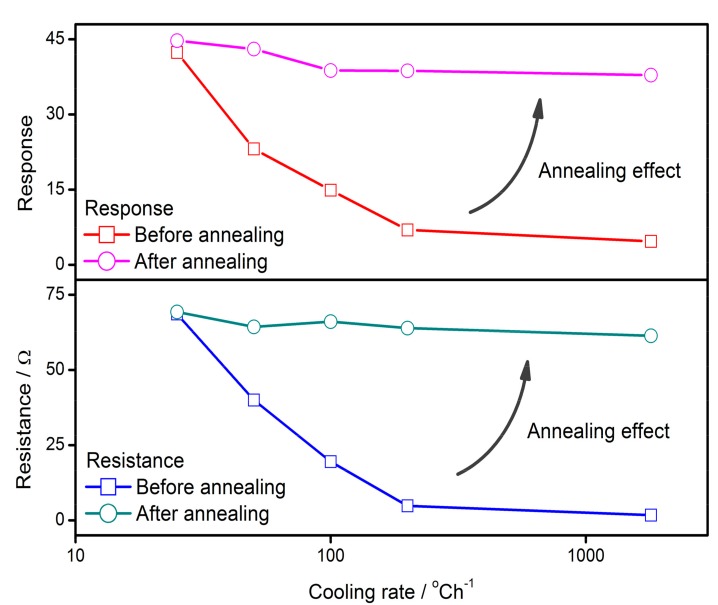
The influences of cooling rate on gas-sensing characteristics of sensor resistance and response to 13.7 ppm of H_2_S at 100 °C, before and after annealing process. This experiment uses the smallest cooling rate of 25 °C/h. The data was extracted from previous references [91,93].

The GDOV model is established on the experimental basis of the influence of cooling rate on the gas-sensing characteristics of SnO_2_ thin films. As described in the previous work [91], the sol-gel spin-coating technique was employed to prepare the SnO_2_ thin films on alumina substrates with silver interdigital electrodes. The thin films were sintered at 550 °C with various cooling rates, which were program controlled. Some of the samples were quenched with a cooling rate of approximately 3600 °C/h. The gas-sensing characteristics of the prepared thin films were measured in a computer-controlled system, which used the reducing H_2_S gas as a stimulant gas. The X-ray diffraction (XRD) patterns of the samples show that the cooling rate has little impact on the film structure and grain size, which is approximately 21 nm according to Scherer’s formula. Some quenched thin films were annealed once again at 550 °C with the smallest cooling rate and no change in film structure was observed based on the XRD patterns [91]. The relationships between cooling rate and gas-sensing characteristics are shown in Figure 1. The sensor response to reducing gas (*S*) is defined as the ratio of film resistance in air (*R_a_*) to the resistance in stimulant gas (*R_g_*), namely *S* = *R_a_*/*R_g_*. The sensor resistance and response to H_2_S have negative correlations with the cooling rate. Both of the sensor performances benefit from the low cooling rate. Each of the thin films with a larger cooling rate was annealed at the initial sintering temperature of 550 °C, before the smallest cooling rate was conducted in all of them. The annealing effects of film resistance and response are also shown in Figure 1, in which the film properties are enhanced to the same level as the one with the smallest cooling rate at the first time. It appears that a process that determines the sensor properties is interrupted by the fast cooling and it is restarted by the subsequent annealing. 

**Figure 2:**
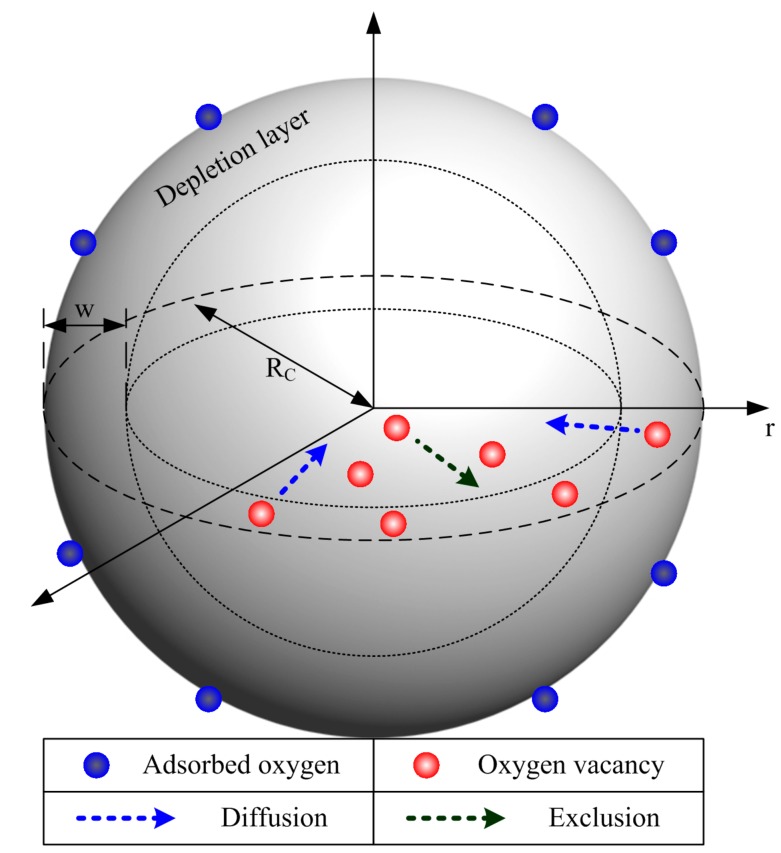
Spherical coordinates in an ideal grain with a radius of *R_C_* and a depletion layer width of *w* with adsorbed oxygen on the grain surface and vacancies inside the grain. These are under control of diffusion and exclusion effects.

This phenomenon drives us to explore the internal mechanism of the semiconductor gas sensor. Several classical theories are employed in the investigation of SnO_2_ samples with the following presumptions: (1) a SnO_2_ thin film consists of tiny grains with uniform inherent oxygen vacancies, which act as donors and provide free electrons after ionization; (2) all free electrons are seized by adsorbed oxygen and no free electron appears in the depletion layer, as mentioned in the abrupt model [97]; and (3) the potential barrier at grain boundaries is described by Poisson’s equation as indicated in the double Schottky barrier model. Mathematically, the sensor resistance (*R*) and response to the reducing gas (*S*) can be formulated as Equations (1) and (2), the derivation of which are described in detail in the previous work [91].

(1) $$R=R_{0}\exp(\frac{q^{2}w^{2}}{\varepsilon kT}N_{V})$$

(2) $$S=\exp(\frac{\alpha q^{2}w^{2}}{\varepsilon kT}N_{V})$$

where *R*_0_ is the resistance under flat-band condition [89]; *q*, *ε* and *k* are the elemental charge of electron, the permittivity of the SnO_2_ material and the Boltzmann constant, respectively; *T* is the operating temperature; *w* is the width of the depletion layer; *α* denotes the percentage of electrons released back to the depletion layer during gas detection; and *N_V_* is the density of effective donors, which is acted on by ionized oxygen vacancies. Equations (1) and (2) indicate that the resistance and response are fundamentally influenced by V_O_, such as its density, distribution and migration. Therefore, a discussion is expected for the kinetics and behaviors of V_O_ in the semiconductor grain during the sintering and cooling process. For convenience in the following discussion, spherical coordinates are established in a tiny grain, which is in ideally spherical shape with the radius of *R_C_* and a depletion layer width of *w*, as shown in Figure 2. Two kinetics of V_O_ are taken into consideration, which are namely diffusion and exclusion. The diffusion takes place when inhomogeneous V_O_ density appears between nearby positions in the grain, following the Fick’s law. It could happen at any time provided that the operating temperature is above absolute zero K. The other one is exclusion, appearing during the cooling process, which provides the excluding trend to the defects during thermal vibration inside a crystallite according to the conclusions in the crystal growth theory [98,99,100]. Therefore, both diffusion and exclusion take place simultaneously during the cooling process. Considering the symmetry of the sphere system, the diffusion equation of V_O_ can be established, as shown in Equation (3).

(3) $$\frac{\partial N_{V}(r,t)}{\partial t}=D_{V}\frac{\partial^{2}N_{V}(r,t)}{\partial r^{2}}-PN_{V}(r,t)$$

where *N_V_*(*r*, *t*) denotes the V_O_ density at a position of *r* and at time of *t*; *D_V_* is the diffusion coefficient and exponential to *E_D_* if temperature is fixed, as described in Equation (4),

(4) $$D_{V}=D_{0}\exp(-\frac{E_{D}}{kT_{E}})$$

where *E_D_* is activated energy of diffusion; *D*_0_ is the pre-exponential constant; and *T_E_* is the temperature that the process carries on in.

It is mentioned that oxygen defects increase the energy of the crystal system and they are provided with a tendency for exclusion during thermal vibration in the cooling process. Thus, *P* indicates the possibility of the exclusion for a defect moving outwards to a nearby position in unit time and it can be formulated as follows:

(5) $$P=\nu_{0}\exp(-\frac{E_{\varphi }-E_{0}}{kT_{E}})$$

where *ν*_0_ is the thermal vibration frequency of the oxygen atom; *E_φ_* represents the activated energy of V_O_ migration; and *E*_0_ is the unit energy decrease of the system that results from the one-step exclusion of defects. 

The solution *N_V_*(*r*, *t*) of the diffusion equation contains two parts, which are namely the steady-state solution, *N_Vst_*(*r*), and the transient-state solution, *N_Vtr_*(*r*, *t*), as shown in Equation (6):

(6) $$N_{V}(r,t)=N_{Vst}(r)+N_{Vtr}(r,t)$$

The boundary conditions and initial condition are assumed to be Equations (7) and (8):

(7) $$\{\begin{array}{c} \frac{\partial N_{V}(r,t)}{\partial r}=0,r=0\\ N_{V}(r,t)=N_{VS},r=R_{C} \end{array}$$

(8) $$N_{V}(r,t)=N_{0},t=0$$

where *N_VS_* is the V_O_ density on the grain surface and *N*_0_ is the uniform density of V_O_ throughout the grain at the beginning of cooling process. Thus, it is possible to find the steady-state and transient-state solutions.

### 2.2. Steady-State Distribution

For the steady-state solution, the diffusion equation of Equation (3) is simplified to Equation (9) because *N_V_*(*r*, *t*) is time-independent at the steady state when the cooling rate is sufficiently small.

(9) $$D_{V}\frac{\partial^{2}N_{Vst}(r)}{\partial r^{2}}=PN_{Vst}(r)$$

Thus, the general solution for Equation (9) can be obtained easily as follows:

(10) $$N_{Vst}(r)=C_{1}\exp(mr)+C_{2}\exp(-mr)$$

(11) $$m=\sqrt{\frac{P}{D_{V}}}=\sqrt{\frac{\nu_{0}}{D_{0}}}\exp(\frac{E_{D}-E_{\varphi }+E_{0}}{2kT_{E}})$$

where *C*_1_ and *C*_2_ are integral constants; *m* is defined in Equation (11). Considering the boundary conditions, which are degraded to Equation (12), the steady-state solution can be found after *C*_1_ and *C*_2_ are calculated.

(12) $$\{\begin{array}{c} \frac{\partial N_{Vst}(r)}{\partial r}=0,r=0\\ N_{Vst}(r)=N_{VS},r=R_{C} \end{array}$$

(13) $$N_{Vst}(r)=\frac{N_{VS}}{\cosh(mR_{C})}\cosh(mr)$$

**Figure 3:**
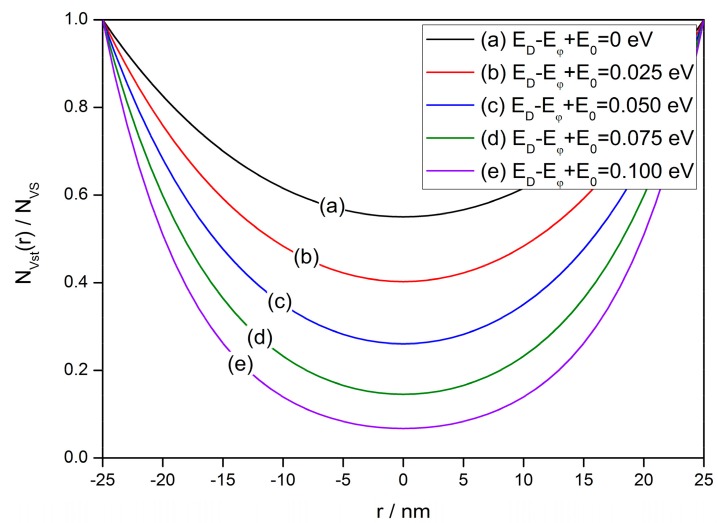
Steady-state distribution of oxygen vacancies in a semiconductor grain with a radius of 25 nm at various values of *E_D_* − *E_φ_* + *E*_0_ from 0 to 0.1 eV, which determines the gradient of the oxygen vacancy density profile.

Hence, the steady-state V_O_ distribution in a semiconductor grain according to Equations (11) and (13) and the profiles in one-dimensional model are shown in Figure 3. This figure reveals that the V_O_ tends to accumulate near the surface, forming a gradient distribution. The gradient of profile is determined by the value of *m*, which is a function of *E_D_*, *E_φ_*, *E*_0_, *D*_0_ and *ν*_0_ when *T_E_* is given. Indeed, *m* indicates the end temperature of the annealing process with a sufficiently low cooling rate. Some of the constants above can be known from previous researches, such as *D*_0_ = 0.0431 m^2^/s, *E_D_* = 2.7 eV [101] and *ν*_0_ = 10^14^ s^−1^ [102]. However, there is no convincing conclusion for *E_φ_* and *E*_0_. It is noted that *E_D_* and *E_φ_* are the activated energy of diffusion and exclusion, which have the same migrating mechanism that an oxygen atom change position with an oxygen vacancy across potential between Sn atoms. Thus, *E_D_* and *E_φ_* would not have values with a large difference. However, *E*_0_ should be much smaller that *E_D_* and *E_φ_* according to the definitions. Hence, it is tolerable to estimate that *E_D_* ≈ *E_φ_* >> *E*_0_ [91]. Their influence on the V_O_ distribution is shown in Figure 3. A larger value of *E_D_* − *E_φ_* + *E*_0_ will provide a greater gradient of V_O_ distribution.

### 2.3. Transitent-State Distribution

The next step is to find the solution at the transient state, *N_Vtr_*(*r*, *t*). The diffusion equation for the transient state can be expressed as Equation (14) after the removal of steady-state solution *N_Vst_*(*r*).

(14) $$\frac{\partial N_{Vtr}(r,t)}{\partial t}=D_{V}\frac{\partial^{2}N_{Vtr}(r,t)}{\partial r^{2}}-PN_{Vtr}(r,t)$$

Hence, the boundary conditions and initial condition are transformed into Equations (15) and (16) accordingly.

(15) $$\{\begin{array}{c} \frac{\partial N_{Vtr}(r,t)}{\partial r}=0,r=0\\ N_{Vtr}(r,t)=N_{VS},r=R_{C} \end{array}$$

(16) $$N_{Vtr}(r,t)=N_{0}-N_{Vst}(r),t=0$$

Assuming that *N_Vtr_*(*r*, *t*) = *f*(*r*)*g*(*t*), where *f*(*r*) and *g*(*t*) are space and time-dependent functions, respectively, the Equation (14) is transformed into Equation (17):

(17) $$\frac{dg(t)}{g(t)dt}=D_{V}\frac{d^{2}f(r)}{f(r)dr^{2}}-P$$

If both sides of Equation (17) are equal to −*λ* (eigenvalue), the general solution of the time-dependent function *g*(*t*) can be easily found as Equation (18). It is noted that *g*(*t*) is convergent when *t* reaches infinite only in the case that *λ* is positive.

(18) g(t)=exp(−λt)

For the space-dependent part, the general solution of *f*(*r*) can be found to be Equation (19) provided that the condition of *λ* − *P* > 0 is met to avoid meaningless solutions of *f*(*r*).

(19) $$f(r)=A\cos(\sqrt{\frac{\lambda -P}{D_{V}}}r)+B\sin(\sqrt{\frac{\lambda -P}{D_{V}}}r)$$

where *A* and *B* are pending constants, which can be found by using the boundary conditions of *f’*(0) = 0 and *f*(*R_C_*) = 0. Thus, the specific solution of *f*(*r*) can be found to be Equation (20), while the eigenvalue *λ* is calculated to be Equation (21).

(20) $$f(r)=\sum_{n=0}^{\infty }A_{n}\cos(\frac{2n+1}{2R_{C}}\pi r)$$

(21) $$\lambda_{n}=P+{\left(\frac{2n+1}{2R_{C}}\right)}^{2}\pi^{2}D_{V}$$

Therefore, the transient-state solution *N_Vtr_*(*r*, *t*) can be obtained, as expressed in Equation (22).

(22) $$N_{Vtr}(r,t)=f(r)g(t)=\sum_{n=0}^{\infty }A_{n}\cos(\frac{2n+1}{2R_{C}}\pi r)\exp(-\lambda_{n}t)$$

The initial condition of Equation (16) is then rewritten as Equation (23) and the pending constants *A_n_* can be found in Equation (24).

(23) $$N_{Vtr}(r,0)=\sum_{n=0}^{\infty }A_{n}\cos(\frac{2n+1}{2R_{C}}\pi r)=N_{0}-N_{Vst}(r)$$

(24) $$A_{n}=[\frac{4N_{0}}{(2n+1)\pi }-\frac{4\pi (2n+1)N_{VS}}{4m^{2}R_{C}^{2}+{\left(2n+1\right)}^{2}\pi^{2}}]{\left(-1\right)}^{n}$$

Therefore, the whole solution of the diffusion equation of Equation (3) is acquired, as Equations (25)–(27).

(25) $$\begin{array}{l} N_{V}(r,t)=N_{Vst}(r)+N_{Vtr}(r,t)\\ =\frac{N_{VS}}{\cosh(mR_{C})}\cosh(mr)+\sum_{n=0}^{\infty }A_{n}\cos(\frac{2n+1}{2R_{C}}\pi r)\exp(-\lambda_{n}t) \end{array}$$

(26) $$A_{n}=[\frac{4N_{0}}{(2n+1)\pi }-\frac{4\pi (2n+1)N_{VS}}{4m^{2}R_{C}^{2}+{\left(2n+1\right)}^{2}\pi^{2}}]{\left(-1\right)}^{n}$$

(27) $$\lambda_{n}=P+{\left(\frac{2n+1}{2R_{C}}\right)}^{2}\pi^{2}D_{V}$$

**Figure 4:**
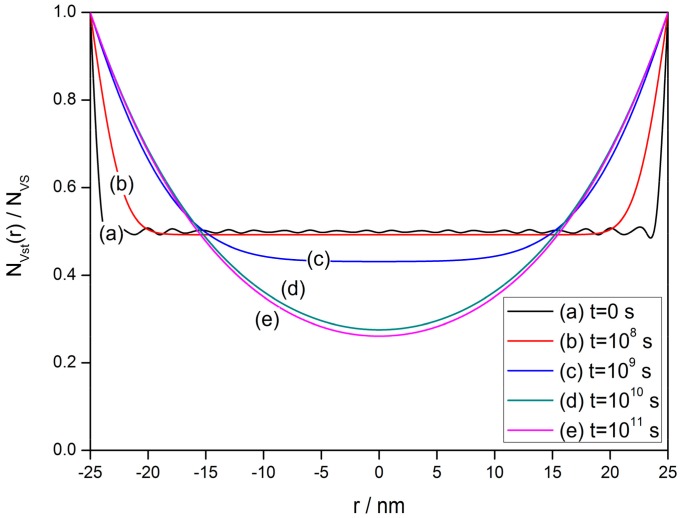
Transient process of oxygen vacancy distribution in a typical semiconductor grain with a radius of 25 nm from the initial uniform distribution (*N*_0_ = 0.5 *N_VS_*) to gradient distribution at a steady state (*t* > 10^11^ s). Constant settings are used as: *N_VS_* = 5 × 10^25^ m^3^, *N*_0_/*N_VS_* = 0.5 and *E_D_* − *E_φ_* + *E*_0_ = 0.05 eV.

The solution reveals the transient V_O_ distribution from the initial uniform stage to the steady state during the ideal cooling process, in which the cooling rate is sufficiently small. Figure 4 shows the transient process of V_O_ distribution in a typical semiconductor grain with radius of 25 nm. The constants are set to be: *N_VS_* = 5 × 10^25^ m^3^ [89], *N*_0_/*N_VS_* = 0.5 [90] and *E_D_* − *E_φ_* + *E*_0_ = 0.05 eV. For convenience in calculation, the first 20 terms of Equations (25)–(27) (*n* = 0–19) are reserved so the vibration appears around *N*_0_/*N_VS_* in curve (a) in Figure 4. During the ideal cooling process, the oxygen vacancies are driven from an initial uniform density to the gradient distribution at a steady state under the effects of diffusion and exclusion.

**Figure 5:**
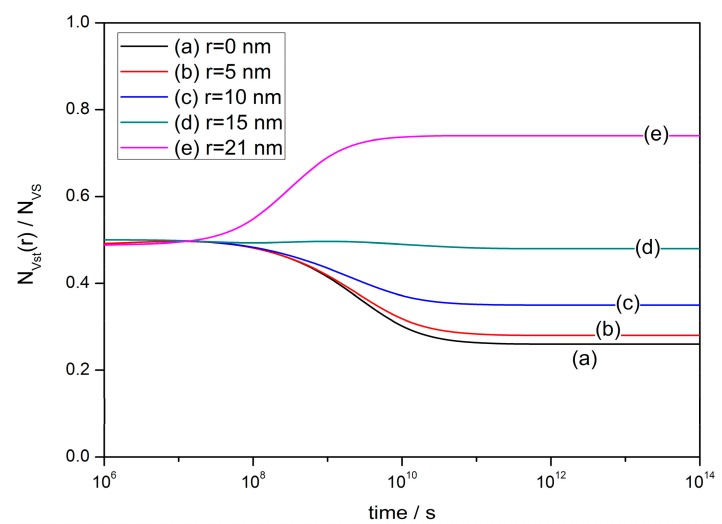
Time-dependent density of oxygen vacancies at various points (r = 0, 5, 10, 15 and 21 nm) in the semiconductor grain with a radius of 25 nm. Constant settings are used as: *N_VS_* = 5 × 10^25^ m^3^, *N*_0_/*N_VS_* = 0.5 and *E_D_* − *E_φ_* + *E*_0_ = 0.05 eV.

Figure 5 shows the time-dependent density of oxygen vacancies at various points in the semiconductor grain with radius of 25 nm, using the same constant settings as Figure 4. The defect density near the grain center descends along with time. However, the V_O_ density increases in the depletion layer, where *r* > 21 nm if *w* is assumed to be 4 nm [103,104]. Thus, the total amount of oxygen vacancies is increasing during the ideal cooling process and it would be responsible for the enhancements of gas-sensing properties of the thin films.

**Figure 6:**
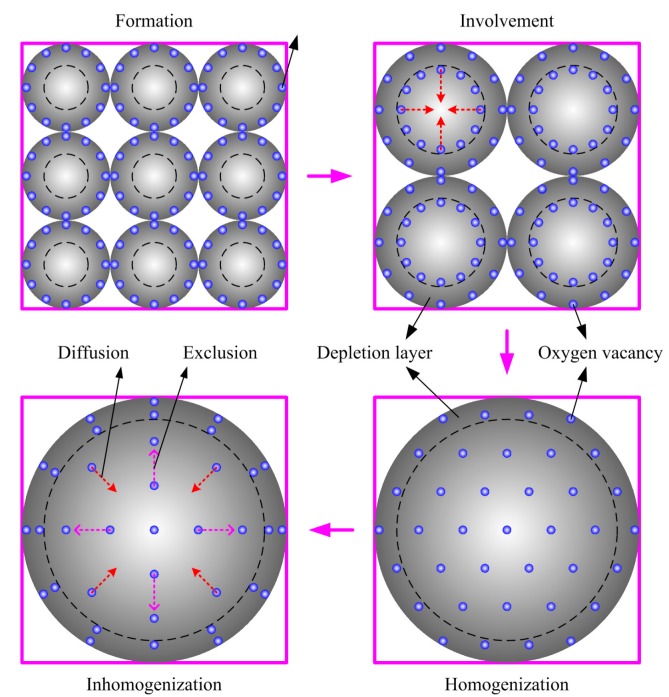
Schematic drawing of the four stages of oxygen vacancy behaviors in semiconductor grains during sintering and cooling process: formation, involvement, homogenization and inhomogenization, under the migration mechanisms of diffusion and exclusion.

The behaviors of oxygen vacancies in semiconductor grains from sintering to cooling process could be divided into four stages, which are namely formation, involvement, homogenization distribution and inhomogenization distribution (Figure 6). At the first stage of sintering, the tiny grains increase in size and oxygen vacancy formation takes place on the grain surface due to the oxygen atoms escaping from the grain lattice to the aerial atmosphere. Along with the growth of grains, the vacancies are involved into the grains and the diffusion effect will drive the defects to distribute homogeneously. Following this, at the end of sintering process, the grain ceases to grow up and the vacancies randomly spread throughout the grain, leaving an approximately homogeneous distribution. Once the cooling process starts, the oxygen vacancies are provided with an excluding tendency. An inhomogeneous V_O_ distribution appears under the synergy of diffusion and exclusion in the cooling process, when oxygen vacancies aggregate near the surface. This procedure could explain why the quenched samples have less resistance and response than the samples with a low cooling rate (Figure 1). The quenched samples have a homogeneous V_O_ distribution because the defects are frozen at the places where they are at the end of sintering. In this case, the density of oxygen vacancies in the depletion layer is the same as the one in the center bulk. However, along with the cooling or annealing process, oxygen defects accumulate in the depletion layer and this accumulation of V_O_ is responsible for the higher gas-sensing characteristics of the thin films.

## 3. Gas-Sensing Characteristics

### 3.1. Sensor Resistance

**Figure 7:**
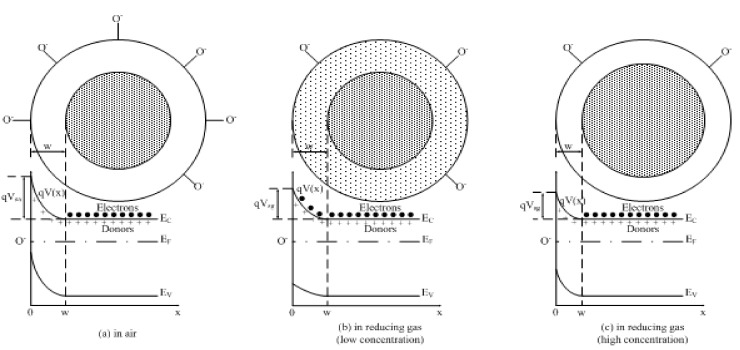
Schematic drawing of an one-dimensional Schottky barrier model and potential energy for electrons: (**a**) in aerial atmosphere; (**b**) in low concentration reducing gas and (**c**) in high concentration reducing gas, where *E_C_*, *E_V_* and *E_F_* are the conduction band energy, valence band energy and Fermi energy, respectively.

It is possible to calculate the gas-sensing characteristics of semiconductor thin films if the V_O_ distribution is successfully formulated. As shown in Figure 7a, a Schottky barrier is established due to the ionized V_O_ accumulation after the free electrons in the depletion layer are seized by adsorbed oxygen on grain surface. It is known that the electric potential in the depletion layer can be described by the Poisson’s equation as follows:

(28) $$\frac{d^{2}V(x)}{dx^{2}}=-\frac{\rho (x)}{\varepsilon },0<x<w$$

where *V*(*x*) denotes the electric potential at given depth; while *ρ*(*x*) is the space charge density, which can be expressed as *ρ*(*x*) = *q*[*N_d_^+^*(*x*) − *n*(*x*)] in n-type semiconductors, where *N_d_^+^*(*x*) and *n*(*x*) are the densities of ionized donors and electrons at a given depth, respectively. Assuming that all electrons in depletion layer are seized by adsorbed oxygen and all oxygen vacancies are first-order ionized, the space charge density *ρ*(*x*) is equal to *N_V_*(*x*), which is the V_O_ density at given depth and can be formulated easily by transforming Equations (25)–(29).

(29) $$N_{V}(x,t)=\frac{N_{VS}}{\cosh(mR_{C})}\cosh[m(x-R_{C})]+\sum_{n=0}^{\infty }A_{n}\cos[\frac{2n+1}{2R_{C}}\pi (x-R_{C})]\exp(-\lambda_{n}t)$$

Thus, the Poisson’s equation is rewritten as Equation (30) provided that the abrupt model is applied [105]. *V*(*x*,*t*) can be found by using the classic boundary conditions of *V*(*w*) = 0 and *V*’(*w*) = 0.

(30) $$\frac{\partial^{2}V(x,t)}{\partial x^{2}}=-\frac{qN_{V}(x,t)}{\varepsilon },0<x<w$$

The potential barrier height, *qV_S_*, is the potential barrier *qV*(*x*, *t*) at the surface (*x* = 0). It is then expressed in Equation (31).

(31) $$\begin{array}{l} qV_{S}(t)=qV(0,t)\\ =\left\{\begin{array}{c} \frac{q^{2}N_{VS}}{\varepsilon m^{2}\cosh(mR_{C})}\{\cosh(mR_{C})-\cosh[m(w-R_{C})]+mw\sinh[m(w-R_{C})]\}\\ +\sum_{n=0}^{\infty }\frac{2q^{2}R_{C}}{{\left(2n+1\right)}^{2}\pi^{2}\varepsilon }A_{n}\left\{\begin{array}{c} \left(2n+1)\pi w\sin[\frac{2n+1}{2R_{C}}\pi (w-R_{C})\right]\\ +2R_{C}\cos[\frac{2n+1}{2R_{C}}\pi (w-R_{C})] \end{array}\right\}\exp(-\lambda_{n}t) \end{array}\right\} \end{array}$$

The sensor resistance (*R*) is usually considered to be exponential to *qV_S_* and it is obtained in Equation (32), where *R*/*R*_0_ is called the reduced resistance.

(32) $$\begin{array}{l} \frac{R(t)}{R_{0}}=\exp[\frac{qV_{S}(t)}{kT}]\\ =\exp\left\{\begin{array}{c} \frac{q^{2}N_{VS}}{\varepsilon m^{2}kT\cosh(mR_{C})}\{\cosh(mR_{C})-\cosh[m(w-R_{C})]+mw\sinh[m(w-R_{C})]\}\\ +\sum_{n=0}^{\infty }\frac{2q^{2}R_{C}}{{\left(2n+1\right)}^{2}\pi^{2}\varepsilon kT}A_{n}\left\{\begin{array}{c} \left(2n+1)\pi w\sin[\frac{2n+1}{2R_{C}}\pi (w-R_{C})\right]\\ +2R_{C}\cos[\frac{2n+1}{2R_{C}}\pi (w-R_{C})] \end{array}\right\}\exp(-\lambda_{n}t) \end{array}\right\} \end{array}$$

**Figure 8:**
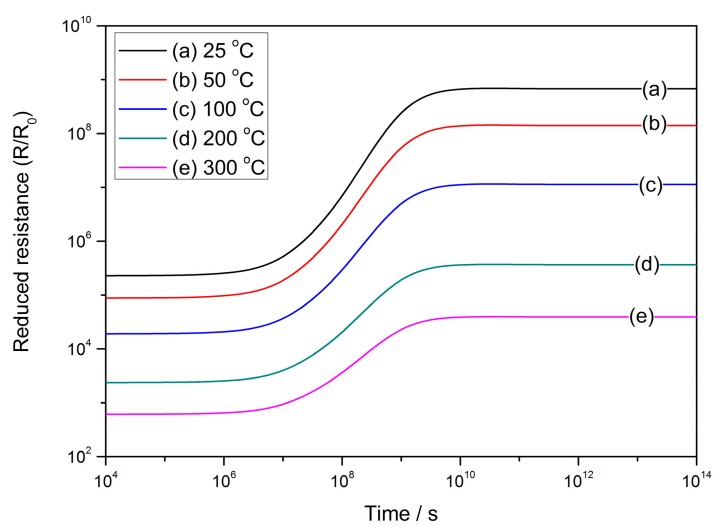
Time-dependent reduced resistance at various operating temperatures from 25 to 300 °C during the ideal cooling process when the constants are set to be: *R_C_* = 25 nm, *N_VS_* = 5 × 10^25^ m^3^, *N*_0_/*N_VS_* = 0.5, *ε* = 10^−10^ F/m, *w* = 4 nm and *E_D_* − *E_φ_* + *E*_0_ = 0.05 eV.

Figure 8 shows the performance of reduced resistance in the ideal cooling process at the operating temperatures of 25–300 °C according to Equation (32). The constants and variables are set to be: *R_C_* = 25 nm, *N_VS_* = 5 × 10^25^ m^3^ [89], *N*_0_/*N_VS_* = 0.5 [90], *ε* = 10^−10^ F/m [106], *w* = 4 nm [103,104] and *E_D_* − *E_φ_* + *E*_0_ = 0.05 eV. Figure 8 infers that it is possible to control the sensor resistance by restricting the time elapsed in the ideal cooling process.

### 3.2. Response to Reducing Gases

When the semiconductor grains are exposed to reducing gases, a part of adsorbed oxygen is consumed by reducing gas molecules. Thus, a certain percentage of seized electrons are released back to the depletion layer. These released electrons are under control from two mechanisms. One is the potential barrier from the accumulation of ionized oxygen vacancies and the electrons tend to locate themselves at the verge of the depletion layer. The other one is the diffusion of electrons and it will counteract the first mechanism. For convenience in discussion, the following calculation of the sensor response is divided into two cases: (1) For the first case with a low concentration of stimulant gas, the density of released electrons is limited. The electrons are assumed to be spread uniformly in the depletion layer. The depletion layer width remains the same in this case. (2) For the other case with a high concentration of stimulant gas, the released electrons primarily compensate the ionized oxygen vacancies near the edge of the depletion layer. Therefore, the depletion layer width is decreased in this case.

#### 3.2.1. Low Gas Concentration

As described in Figure 7b, the release electrons spread uniformly in the depletion layer. If *n_R_* denotes the density of released electrons, the Poisson’s equation, as shown in Equation (30), changes to Equation (33).

(33) $$\frac{\partial^{2}V(x,t)}{\partial x^{2}}=-\frac{q[N_{V}(x,t)-n_{R}]}{\varepsilon },0<x<w$$

Following this, the sensor response (*S*) in Equation (34) is obtained from the film resistance in air (*R_a_*), which is already expressed in Equation (32), as well as the resistance in stimulant gas (*R_g_*), which is found after Equation (33) is solved. 

(34) $$S=\frac{R_{a}}{R_{g}}=\frac{\exp(\frac{qV_{Sa}}{kT})}{\exp(\frac{qV_{Sg}}{kT})}=\exp(\frac{q^{2}w^{2}}{2\varepsilon kT}n_{R})$$

If a semiconductor grain is placed in an atmosphere without oxygen, no adsorbed oxygen would exist on the surface and no electron is seized. Considering that electrons can migrate freely in the grain, *n_w_* denotes the uniform electron density in the imaginary layer with the depth of *w* from surface. Its value is constant for a specific sample at a given temperature. The coefficient *α*, defined as the ratio of *n_R_* and *n_w_*, represents the proportion of the seized electrons that are released back to depletion layer after gas exposure. This coefficient, *α* has a value from 0 (in air) to 1 (in vacuum or other atmosphere free of oxygen), which is correlated with the partial pressure of oxygen and reducing gas. With the presumption of first-order ionization of oxygen vacancy, Equation (35) is acquired because all free electrons come from the ionized oxygen vacancies.

(35) $$n_{R}=\alpha n_{w}=\alpha \bar{N_{V}}$$

where $\bar{N_{V}}$ is the average V_O_ density throughout the grain. Thus, *n_R_* and *S* can be formulated in Equations (36) and (37).

(36) $$n_{R}=\alpha \bar{N_{V}}=\frac{\alpha }{R_{C}}\int_{0}^{R_{C}}N_{V}(x,t)dx=\frac{\alpha N_{VS}}{mR_{C}}\tanh(mR_{C})+\sum_{n=0}^{\infty }\frac{{\left(-1\right)}^{n}2\alpha }{(2n+1)\pi }A_{n}\exp(-\lambda_{n}t)$$

(37) $$S=\exp\{\frac{q^{2}w^{2}}{\varepsilon kT}[\frac{\alpha N_{VS}}{2mR_{C}}\tanh(mR_{C})+\sum_{n=0}^{\infty }\frac{{\left(-1\right)}^{n}\alpha }{(2n+1)\pi }A_{n}\exp(-\lambda_{n}t)]\}$$

**Figure 9:**
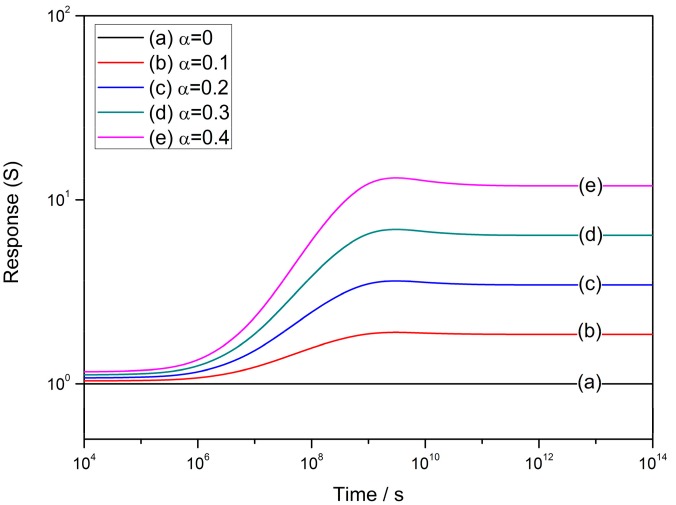
Time-dependent sensor response to reducing gas with low concentration at various *α* values of 0–0.4 at the operating temperature of 300 °C, where the constants are set to be: *R_C_* = 25 nm, *N_VS_* = 5 × 10^25^ m^3^, *N*_0_/*N_VS_* = 0.5, *ε* = 10^−10^ F/m, *w* = 4 nm, *T* = 573 K and *E_D_* − *E_φ_* + *E*_0_ = 0.05 eV.

The sensor responses to reducing gas with low concentrations at various *α* values are shown in Figure 9, in which the constants and valuables are set to be: *R_C_* = 25 nm, *N_VS_* = 5 × 10^25^ m^3^ [89], *N*_0_/*N_VS_* = 0.5 [90], *ε* = 10^−^^10^ F/m [106], *w* = 4 nm [103,104], *T* = 573 K and *E_D_* − *E_φ_* + *E*_0_ = 0.05 eV. It is observed that the response increases along with the time elapsed in the ideal cooling process and experiences a peak before it reaches the steady state. However, the peak is not observed in the performance of resistance and the reason for the difference is still unknown.

#### 3.2.2. High Gas Concentration

When the high gas concentration is used, the released electrons will primarily compensate the ionized V_O_ from the verge of depletion layer, as shown in Figure 7c. If the depletion layer widths before and after gas exposure are denoted by *w_a_* and *w_g_* respectively, the following correlation is obtained in the one-dimensional model, as Equation (38).

(38) $$w_{g}=(1-\alpha )w_{a}$$

Therefore, the corresponding *R_a_* and *R_g_* can be easily transformed from Equation (32) to Equations (39) and (40), which are used to calculate *S* and its time dependence by *S* = *R_a_*/*R_g_*.

(39) $$\frac{R_{a}}{R_{0}}=\exp\left\{\begin{array}{c} \frac{q^{2}N_{VS}}{\varepsilon m^{2}kT\cosh(mR_{C})}\{\cosh(mR_{C})-\cosh[m(w_{a}-R_{C})]+mw\sinh[m(w_{a}-R_{C})]\}\\ +\sum_{n=0}^{\infty }\frac{2q^{2}R_{C}}{{\left(2n+1\right)}^{2}\pi^{2}\varepsilon kT}A_{n}\left\{\begin{array}{c} \left(2n+1)\pi w_{a}\sin[\frac{2n+1}{2R_{C}}\pi (w_{a}-R_{C})\right]\\ +2R_{C}\cos[\frac{2n+1}{2R_{C}}\pi (w_{a}-R_{C})] \end{array}\right\}\exp(-\lambda_{n}t) \end{array}\right\}$$

(40) $$\frac{R_{g}}{R_{0}}=\exp\left\{\begin{array}{c} \frac{q^{2}N_{VS}}{\varepsilon m^{2}kT\cosh(mR_{C})}\{\cosh(mR_{C})-\cosh[m(w_{g}-R_{C})]+mw\sinh[m(w_{g}-R_{C})]\}\\ +\sum_{n=0}^{\infty }\frac{2q^{2}R_{C}}{{\left(2n+1\right)}^{2}\pi^{2}\varepsilon kT}A_{n}\left\{\begin{array}{c} \left(2n+1)\pi w_{g}\sin[\frac{2n+1}{2R_{C}}\pi (w_{g}-R_{C})\right]\\ +2R_{C}\cos[\frac{2n+1}{2R_{C}}\pi (w_{g}-R_{C})] \end{array}\right\}\exp(-\lambda_{n}t) \end{array}\right\}$$

**Figure 10:**
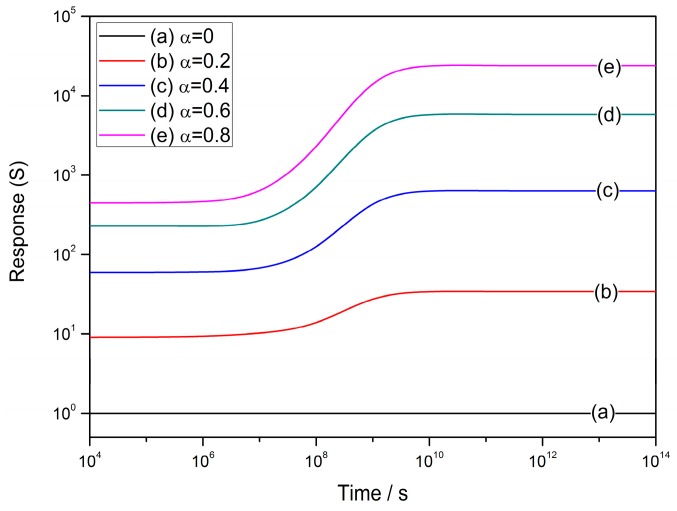
Time-dependent sensor response to reducing gas with high gas concentration at various *α* values of 0–0.8 at the operating temperature of 300 °C, where the constants are set to be: *R_C_* = 25 nm, *N_VS_* = 5 × 10^25^ m^3^, *N*_0_/*N_VS_* = 0.5, *ε* = 10^−10^ F/m, *w* = 4 nm, *T* = 573 K and *E_D_* − *E_φ_* + *E*_0_ = 0.05 eV.

Figure 10 shows the sensor responses to reducing gas with high concentration at various *α* values of 0–0.8. The constants and variables are set to be: *R_C_* = 25 nm, *N_VS_* = 5 × 10^25^ m^3^ [89], *N*_0_/*N_VS_* = 0.5 [90], *ε* = 10^−10^ F/m [106], *w* = 4 nm [103,104], *T* = 573 K and *E_D_* − *E_φ_* + *E*_0_ = 0.05 eV. Unlike Equation (37), the response to reducing gas with high concentration increases monotonously with time elapsed in the ideal cooling process. The cooling process enhances the response and the enhancement is more obvious when the gas concentration is higher. Figure 8, Figure 9, Figure 10 illustrate how the gas-sensing characteristics perform during the ideal cooling process. We can conclude that the sensor properties could be controlled by interrupting the cooling process at a proper time, in order to acquire a gas sensor with required characteristics. For an existing sensor, such as a quenched thin film with low sensor performances, its properties could also be adjusted by a designed annealing process, which brings a redistribution of oxygen vacancies to provide new sensor performances without changing its microstructure. The simulations above may also provide potential explanations to the mechanism of refreshment of gas sensors in practical usage.

### 3.3. Model Validity

**Figure 11:**
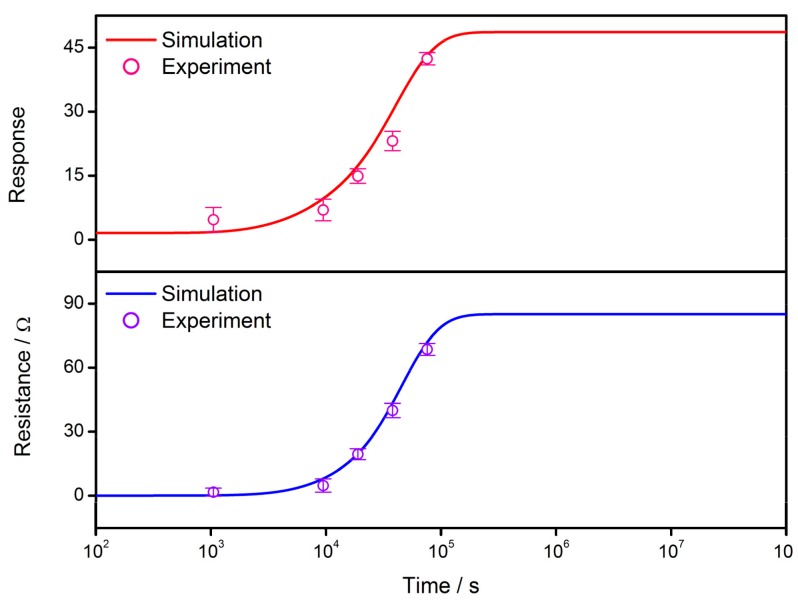
Correlation between simulated gas-sensing characteristics and the performances of actual SnO_2_ thin film gas sensor samples, using data extracted from a previous reference [90]. The constants in simulation are set to be: *R_C_* = 10 nm, *N_VS_* = 5 × 10^25^ m^3^, *N*_0_/*N_VS_* = 0.5, *ε* = 10^−10^ F/m, *w* = 4 nm, *T* = 373 K, *E_D_* − *E_φ_* + *E*_0_ = 0.05 eV, *R*_0_ = 7 × 10^5^ Ω and *α* = 0.12.

As discussed above, the film resistance and response to reducing gases are successfully simulated based on the solutions of the diffusion equation as shown in Equation (3). It is necessary to check the validity of the expressions by correlating them with experimental results. Using the gas sensor prepared by SnO_2_ thin films as previously described [91], the actual gas sensor properties of resistance and response are plotted in Figure 11. It is observed that the simulations are in good agreement with experimental results when the constants and variables are set to be: *R_C_* = 10 nm, *N_VS_* = 5 × 10^25^ m^3^, *N*_0_/*N_VS_* = 0.5, *ε* = 10^−10^ F/m, *w* = 4 nm, *T* = 373 K, *E_D_* − *E_φ_* + *E*_0_ = 0.05 eV, *R*_0_ = 7 × 10^5^ Ω and *α* = 0.12. Although the actual conditions of the cooling process are different from the ideal one, the consistency in Figure 11 proves the validity of GDOV model in explaining the variation mechanism of gas-sensing characteristics during the cooling process.

## 4. Applications in Sensor Performance Simulation

### 4.1. The Power Law

The power law is a unique feature of semiconductor gas sensors. It describes the relationship between the sensor resistance/response and concentration of the stimulant gas. The law was first concluded by Morrison [69] and recently explained in a mathematical manner by Yamazoe [89]. The power law exponent, *n*, is also called sensitivity, which is defined as the slope of response against gas concentration in the logarithmic coordinates, as *n* = dln*S*/dln*P_G_*, where *P_G_* is the partial pressure of the reducing stimulant gas. The present GDOV model can be used to simulate the power law of semiconductor gas sensors. For convenience in discussion, only the steady-state part is taken into consideration in the following calculation, which starts from the expressions of response in Equations (41) and (42) under low and high concentrations, respectively.

(41) $$S=\exp\{\frac{\alpha q^{2}w^{2}N_{VS}}{\varepsilon mR_{C}kT}\tanh(mR_{C})\}$$

(42) $$S=\frac{R_{a}}{R_{g}}=\exp\left\{\frac{q^{2}N_{VS}}{\varepsilon m^{2}kT\cosh(mR_{C})}\left\{\begin{array}{c} 2\sinh\frac{\alpha mw_{a}}{2}\left\{\begin{array}{c} mw_{a}\cosh\frac{m[(2-\alpha )w_{a}-2R_{C}]}{2}\\ -\sinh\frac{m[(2-\alpha )w_{a}-2R_{C}]}{2} \end{array}\right\}\\ +\alpha mw_{a}\sinh\{m[(1-\alpha )w_{a}-R_{C})]\} \end{array}\right\}\right\}$$

In order to find the relationship between *S* and *P_G_*, we need to establish the function of *α* against *P_G_*. If O^−^ is assumed to be the only type of adsorbed oxygen, the gas detection procedure could be expressed by the following reactions:

(43) $$\frac{1}{2}O_{2}+e^{-}\overset{k_{1}}{\underset{k_{-1}}{↔}}O^{-}$$

(44) $$G+O^{-}\overset{k_{2}}{\to }GO+e^{-}$$

where the symbol G represents the molecule of reducing gas; *k*_1_, *k_−_*_1_ and *k*_2_ are reaction constants. At the steady state, the reactions reach the equivalence state and Equation (45) is obtained.

(45) $$k_{1}P_{O_{2}}{}^{1/2}[e^{-}]=k_{-1}[O^{-}]+k_{2}P_{G}[O^{-}]$$

where *P_O_*_2_ is the partial pressure of oxygen; while [e^−^] and [O^−^] are the concentrations of the corresponding species, respectively. Actually, [e^−^] is equal to *n_R_*_,_ while *n_w_* is the sum of [O^−^] and *n_R_*. Considering *α* = *n_R_*/*n_w_*, the relationship between *α* and *P_G_* could be found, as Equation (46).

(46) $$\alpha =1-\frac{k_{1}P_{O_{2}}{}^{1/2}}{k_{1}P_{O_{2}}{}^{1/2}+k_{-1}+k_{2}P_{G}}$$

Let *c*_1_ = 1 + *k*_−1_/*k*_1_*P_O_*_2_^1/2^ and *c*_2_ = *k*_2_/*k*_1_*P_O_*_2_^1/2^, so Equation (47) is obtained.

(47) $$\alpha =1-\frac{1}{c_{1}+c_{2}P_{G}}$$

**Figure 12:**
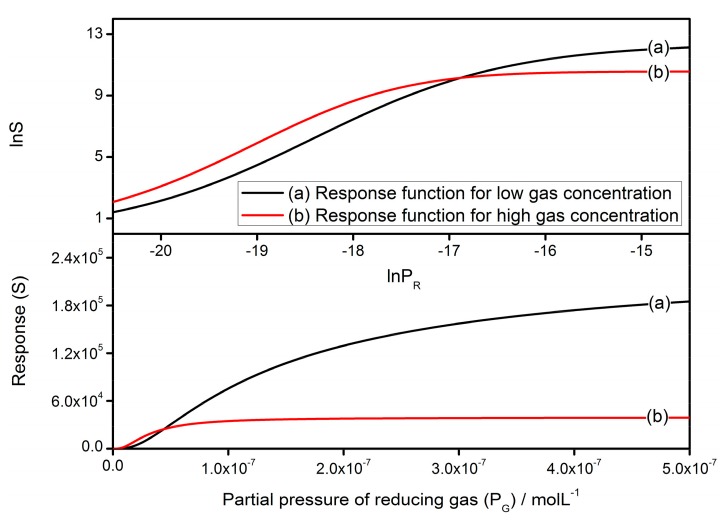
The correlation between sensor response and partial pressure of reducing gas in linear and logarithmic coordinates, where the constants are set to be: *R_C_* = 25 nm, *N_VS_* = 5 × 10^25^ m^3^, *N*_0_/*N_VS_* = 0.5, *ε* = 10^−10^ F/m, *w* = 4 nm, *T* = 573 K, *E_D_* − *E_φ_* + *E*_0_ = 0.05 eV and *α* = 0.4.

Therefore, the correlation between *S* and *P_G_* is found by combining Equations (41), (42) and (47). Figure 12 shows the correlations in both linear and logarithmic coordinates. The constants and variables are set to be: *R_C_* = 25 nm, *N_VS_* = 5 × 10^25^ m^3^ [89], *N*_0_/*N_VS_* = 0.5 [90], *ε* = 10^−10^ F/m [106], *w* = 4 nm [103,104], *T* = 573 K, *E_D_* − *E_φ_* + *E*_0_ = 0.05 eV and *α* = 0.4. In the linear coordinates, both of the response functions perform similarly at first and they reach the saturation when the gas concentration increases. Comparatively, Equation (42) should be more accurate at the high gas concentration region due to the presumption for Equation (41) possibly not being valid at this time. In the logarithmic coordinates, the slopes of curves represent the power law exponent and the sensitivity of sensors. It is observed that the sensitivity remains mostly constant at the low *P_G_* region and is attenuated by the high *P_G_*. This simulation coincides with the theory proposed in Morrison’s and Yamazoe’s works [69,89], which have already explained the phenomenon theoretically.

### 4.2. The Grain Size Effect

**Figure 13:**
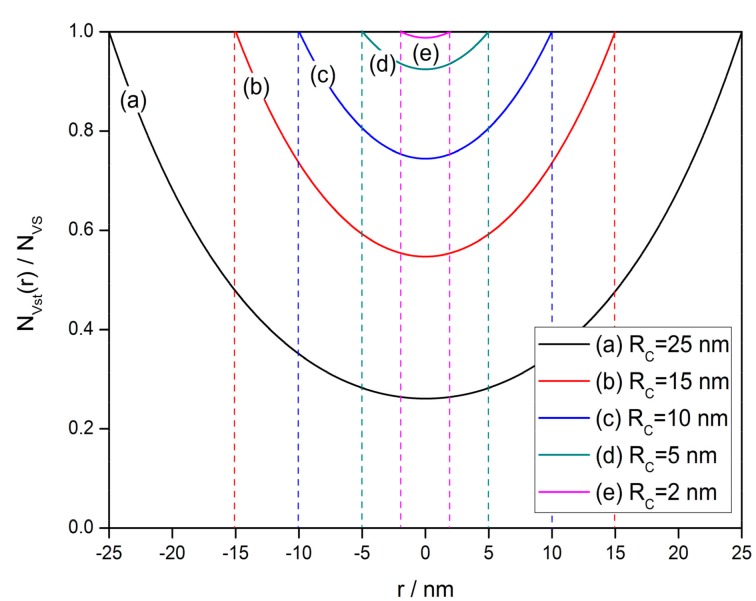
Grain size effect of the oxygen vacancy density distribution in semiconductor grains with radii of 2–25 nm. This indicates that there is only a gap of 1.3% in oxygen vacancy density throughout the grain when the grain radius decreases to 2 nm.

The grain size effect is another important characteristic of semiconductor gas sensors, which summarizes the relationship between the grain size and gas-sensing properties. It was first reported by Xu [107], who proposed a neck-controlled conduction model to explain the dramatic enhancement in resistance and response when the grain radius decreases to the depletion layer width. Figure 13 illustrates the grain size effect of the oxygen vacancy density distribution profile in semiconductor grains with radii of 2–25 nm. A larger grain could maintain a greater density difference between surface and center of the grain. When the grain radius decreases to 2 nm, there is only a gap of 1.3% in V_O_ density throughout the grain. If the depletion layer width is considered to be 3–4 nm [103,107], it means that the V_O_ density is almost homogeneous in the volume-depleted semiconductor grain.

The present GDOV model expresses the sensor resistance and response as functions of grain size. Thus, it is possible to discuss the grain size effect by using the expressions of Equations (48) and (49), which are derived from Equations (32) and (42) if only steady-state solutions are considered.

(48) $$\frac{R}{R_{0}}=\exp\left\{\frac{q^{2}N_{VS}}{\varepsilon m^{2}kT\cosh(mR_{C})}\left\{\begin{array}{c} \cosh(mR_{C})\\ -\cosh[m(w_{a}-R_{C})]\\ +mw_{a}\sinh[m(w_{a}-R_{C})] \end{array}\right\}\right\}$$

(49) $$S=\exp\left\{\frac{q^{2}N_{VS}}{\varepsilon m^{2}kT\cosh(mR_{C})}\left\{\begin{array}{c} 2\sinh\frac{\alpha mw_{a}}{2}\left\{\begin{array}{c} mw_{a}\cosh\frac{m[(2-\alpha )w_{a}-2R_{C}]}{2}\\ -\sinh\frac{m[(2-\alpha )w_{a}-2R_{C}]}{2} \end{array}\right\}\\ +\alpha mw_{a}\sinh\{m[(1-\alpha )w_{a}-R_{C}]\} \end{array}\right\}\right\}$$

**Figure 14:**
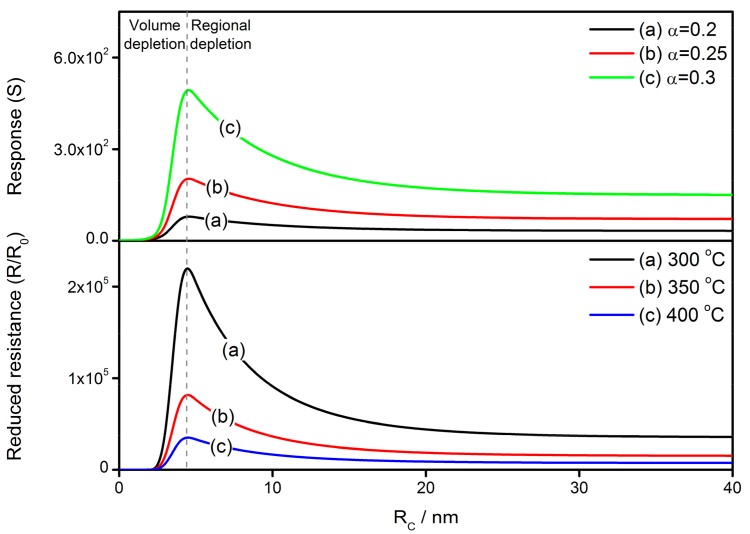
Grain size effects on the gas-sensing characteristics of reduced resistance (*R*/*R*_0_) and response to reducing gas (*S*) at various operating temperatures and gas concentrations in case of regional and volume depletion. The constants are set to be: *N_VS_* = 5 × 10^25^ m^3^, *N*_0_/*N_VS_* = 0.5, *ε* = 10^−10^ F/m, *w* = 4 nm, and *E_D_* − *E_φ_* + *E*_0_ = 0.05 eV.

Thus, the grain size effects on the gas-sensing characteristics of reduced resistance and response to reducing gas are simulated in Figure 14. The constants and variables are set to be: *N_VS_* = 5 × 10^25^ m^3^ [89], *N*_0_/*N_VS_* = 0.5 [90], *ε* = 10^−10^ F/m [106], *w* = 4 nm [103,104], and *E_D_* − *E_φ_* + *E*_0_ = 0.05 eV. As expected, the simulations have an obvious increase when *R_C_* reduces to *w*, with the appearance of the grain size effect in the grain of regional depletion. Once the grain reaches volume depletion, Equations (48) and (49) cease to be valid because the whole grain is depleted. At this time, the width of depletion layer is equal to grain radius, resulting in Equations (50) and (51) being applicable for the grain size effect at the circumstance of volume depletion. The simulations are also illustrated in Figure 14 with the same parameter settings.

(50) $$\frac{R}{R_{0}}=\exp\left\{\frac{q^{2}N_{VS}}{\varepsilon m^{2}kT\cosh(mR_{C})}\{\cosh(mR_{C})-1\}\right\}$$

(51) $$S=\exp\left\{\frac{q^{2}N_{VS}}{\varepsilon m^{2}kT\cosh(mR_{C})}\left\{\begin{array}{c} 2\sinh\frac{\alpha mR_{C}}{2}(mR_{C}\cosh\frac{\alpha mR_{C}}{2}+\sinh\frac{\alpha mR_{C}}{2})\\ -\alpha mR_{C}\sinh(\alpha mR_{C}) \end{array}\right\}\right\}$$

**Figure 15:**
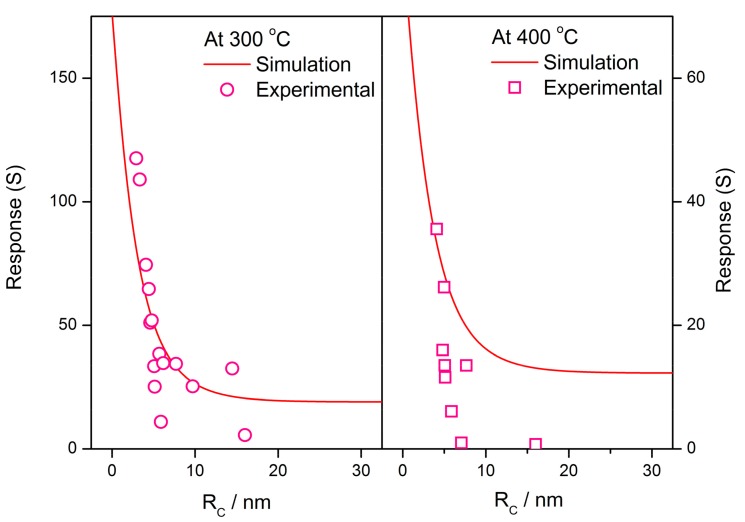
Simulation of grain size effect on gas-sensing characteristics and the comparisons between simulation and experimental results, using data extracted from a previous reference [107]. The constants are set to be: *N_VS_* = 5 × 10^25^ m^3^, *N*_0_/*N_VS_* = 0.5, *ε* = 10^−10^ F/m, *w* = 4 nm, *α* = 0.2 and *E_D_* − *E_φ_* + *E*_0_ = 0.05 eV.

It is imaginable that *R*/*R*_0_ decreases sharply to unity in the case of volume depletion. In this instance, *R*_0_, representing the flat band resistance, becomes much larger because all electrons in the grain are seized by adsorbed oxygen. Actually, the film resistance *R* reaches a very large value and a small *R*/*R*_0_ infers a low potential barrier height between grains, which limits the drop scope by itself during the gas detection. At the same time, the adsorption of oxygen is restricted due to shortage of electron supply in grains. The smaller amount of adsorbed oxygen on grain surface is responsible for a decrease in response to reducing gas. Some conclusions in previous literatures are employed to make comparisons between the simulation and experimental results, as shown in Figure 15. The simulations describe the grain size effect of the response to reducing gas at 300 and 400 °C. The constants and variables are set to be: *N_VS_* = 5 × 10^25^ m^3^ [89], *N*_0_/*N_VS_* = 0.5 [90], *ε* = 10^−10^ F/m [106], *w* = 4 nm [103,104], *α* = 0.2 and *E_D_* − *E_φ_* + *E*_0_ = 0.05 eV. The experimental results are extracted from C. Xu’s report [107], which shows the grain size effect of SnO_2_-based gas sensors. The experimental plots are fitted well by the simulations, especially at the operating temperature of 300 °C.

### 4.3. Effect of Depletion Layer Width

**Figure 16:**
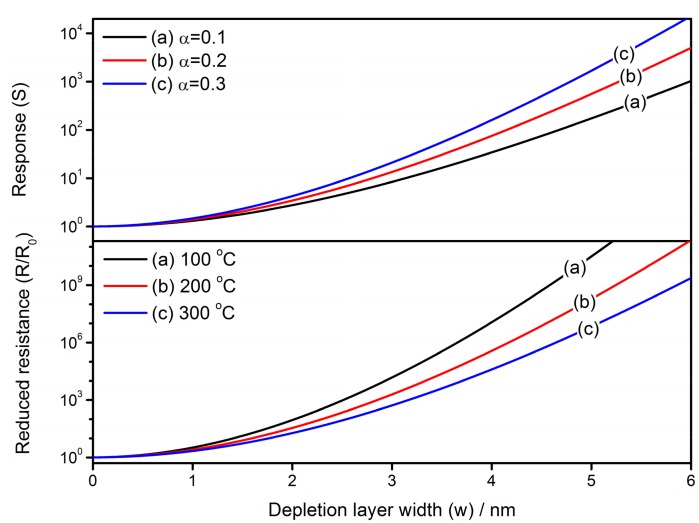
Effects of depletion layer width on gas-sensing characteristics of reduced resistance (*R*/*R*_0_) and response to reducing gas (*S*), in which the constants are set to be: *N_VS_* = 5 × 10^25^ m^3^, *N*_0_/*N_VS_* = 0.5, *ε* = 10^−10^ F/m, *R_C_* = 25 nm and *E_D_* − *E_φ_* + *E*_0_ = 0.05 eV.

**Figure 17:**
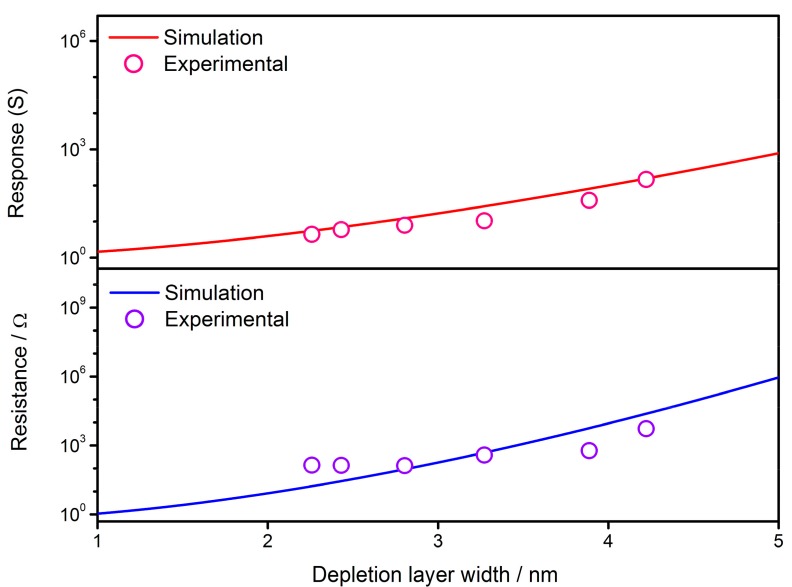
Fitting of simulated gas-sensing properties of resistance and response to reducing gas with experimental results of SnO_2_ thin film gas sensors, using data extracted from a previous reference [103]. The constants in simulation are set to be: *N_VS_* = 5 × 10^25^ m^3^, *N*_0_/*N_VS_* = 0.5, *ε* = 10^−10^ F/m, *R_C_* = 11 nm, *T* = 573 K, *α* = 0.5, *R*_0_ = 0.5 Ω and *E_D_* − *E_φ_* + *E*_0_ = 0.05 eV.

Another relevant topic is the effect of depletion layer width on the gas-sensing characteristics of semiconductor gas sensors. As shown in Figure 16, the reduced resistance (*R*/*R*_0_) and response to reducing gas (*S*) increase along with the expansion of depletion layer in a semiconductor grain. The constants and variables are set to be: *N_VS_* = 5 × 10^25^ m^3^ [89], *N*_0_/*N_VS_* = 0.5 [90], *ε* = 10^−^^10^ F/m [106], *R_C_* = 25 nm and *E_D_* − *E_φ_* + *E*_0_ = 0.05 eV. The comparison is also conducted in Figure 17 by employing experimental results [103], which originate from the SnO_2_ thin films with Sb additive for the control of depletion layer width. The simulation uses the parameter settings as: *N_VS_* = 5 × 10^25^ m^3^ [89], *N*_0_/*N_VS_* = 0.5 [90], *ε* = 10^−^^10^ F/m [106], *R_C_* = 11 nm [103], *T* = 573 K, *α* = 0.5, *R*_0_ = 0.5 Ω and *E_D_* − *E_φ_* + *E*_0_ = 0.05 eV. The simulated gas-sensing properties are in good agreement with the experimental sensor performances, proving the good applicability of the GDOV model for simulating the performances of semiconductor gas sensors.

## 5. Discussion

The inhomogeneous oxygen vacancy distribution in tin oxide grains is discussed based on the influences of cooling rate on the gas-sensing characteristics of SnO_2_ thin films. The variation mechanism of gas sensor performances is explained by the proposed GDOV model, which reveals that the oxygen vacancy behaviors are found to be responsible for the enhancement of gas-sensing properties during cooling and annealing process. The GDOV model describes the steady-state and transient-state distribution of oxygen vacancies, which furthermore formulates the sensor resistance and response to reducing gas. The model is validated by the experimental results and is successfully applied to several circumstances in simulating the properties of gas sensors. However, for convenience in calculation, there are several presumptions and ideal conditions in the discussions above. It is necessary to indicate the reservations of conclusions for further investigations. 

Firstly, it is known that the gas-sensing characteristics of a gas sensor are determined by three factors, which are namely receptor function, transducer function and utility factor. The last one describes how the response attenuates in the sensor body due to the inside gas diffusion. It formulates the sensor resistance and response as functions of the operating temperature, size of grains and pores, gas concentration and thickness of sensing body. Therefore, the sensor performances may appear differently if the utility factor is taken into consideration. However, the present GDOV model focuses on the V_O_ behaviors in a single grain and resistivity at boundary between grains, which are within the scope of receptor function and transducer function, respectively. It is still possible to combine the present model with the utility factor, which is based on the gas diffusion theory, because the dependence of grain resistance on gas concentration is available in Equations (41), (42) and (47). Thus, the connection is established to provide potential entire gas-sensing mechanism for semiconductor gas sensors. Furthermore, the one-dimensional model is used in discussions and amendments may be made when 2D and 3D model are discussed by inserting additional terms in the calculations [108,109]. Secondly, the diffusion equation of Equation (3) is established on the presumption of ideal cooling process, in which the cooling rate is sufficiently small. In this ideal situation, the variable of *T_E_* is used to indicate the end temperature of the cooling process. However, it is very difficult to find the ideal cooling process in practice and this will lead to the deviation of the simulated results from the experimental results. However, it seems impossible to acquire the actual values of *T_E_* in the experimental practice. Hence, the variable *m* has to be treated as a constant in the simulations. Fortunately, *m* is found to be insensitive to the temperature in the concerned range of 25–550 °C [91]. Thus, it is a tolerable approximation of a constant *m* in the simulations. Indeed, *T_E_* is also a time-dependent variable that can be expressed as *T_E_* = *βt*, where *β* and *t* are the cooling rate and time elapsed in the cooling process, respectively. Therefore, an improved diffusion equation that is applicable for any cooling rate may be established by substituting *βt* for *T_E_*, as follows:

(52) $$\frac{\partial N_{V}(r,t)}{\partial t}=D_{0}\exp(-\frac{E_{D}}{k\beta t})\frac{\partial^{2}N_{V}(r,t)}{\partial r^{2}}-\nu_{0}\exp(-\frac{E_{\varphi }-E_{0}}{k\beta t})N_{V}(r,t)$$

**Figure 18:**
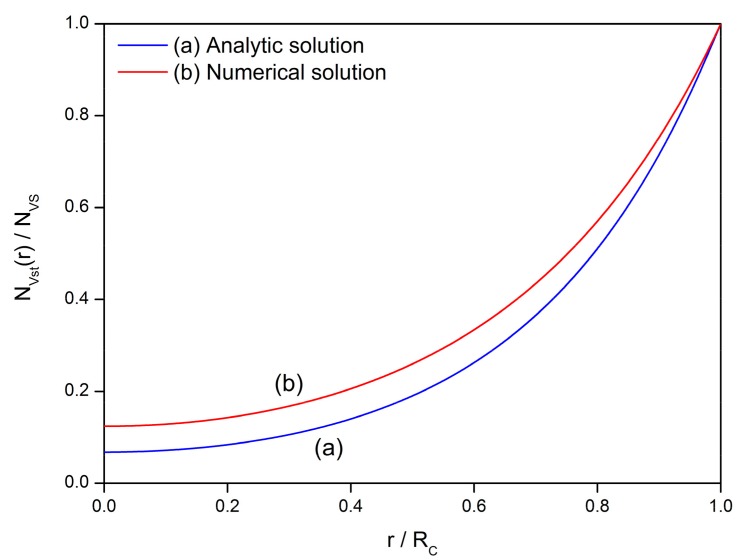
Comparison between analytical solution and numerical solution for the gradient distribution of oxygen vacancies in the semiconductor grain. Numerical solution is calculated by Matlab according to Equation (52).

However, there is no analytical solution for this equation and thus an attempt in numerical analysis is conducted to find the V_O_ distribution profile in the semiconductor grain. Figure 18 reveals the difference between the analytical solution of Equation (3) and the numerical solution of Equation (52). The constants and variables are set to be: *R_C_* = 25 nm, *E_D_* − *E_φ_* + *E*_0_ = 0.1 eV and *β* = 1 K/h. It is observed that both distribution profiles show the same tendency inside a 25-nm grain. However, the numerical solution holds larger values of V_O_ density than the analytical solution with a maximum difference of 6% for *N_VS_*. The deviation is caused by the difference between the ideal cooling process with sufficient low cooling rate and the practical situation with cooling rate of *β* = 1 K/h. Further investigation on the numerical analysis is expected to provide a better simulation for the performances of semiconductor gas sensors. In addition, the setting of parameters needs more discussions. Many parameters are used in the simulation by the GDOV model. The values of them are crucial to the results, some of which are rather sensitive to the parameter settings. Several parameters are universal constants, such as *q* and *k*, or can be experimentally acquired, such as *E_D_*, *w*, *T* and *ε*. However, others are still controversial. In order to continue the discussion, some results and assumptions in previous works have to be used in the calculation, such as *E_φ_* and *E*_0_. The last one is the V_O_ density on surface (*N_VS_*), which is treated as constant in simulation and correlation. Actually, *N_VS_* is a variable that dependent on time, temperature and partial pressure of aerial oxygen, according to Equation (53).

(53) $$O_{O}^{\times }↔V_{O}^{\times }+\frac{1}{2}O_{2}$$

*N_VS_* could increase by the exclusion of *V_O_* in cooling process but the increase could be eliminated by the reversible reaction. A successive detailed investigation on the behaviors of oxygen vacancy on the grain surface is expected for a better explanation of the gas-sensing mechanism of semiconductor gas sensors.

## 6. Conclusions

The oxygen vacancy plays a crucial role in the semiconductor gas sensor, especially in determining sensor performances. The influences of cooling rate on the gas-sensing characteristics are concluded and the conclusion infers that there is an inhomogeneous density distribution of oxygen vacancies in the grains. Thus, the GDOV model is proposed to explain the gas-sensing mechanism by interpreting the vacancy behaviors of formation, involvement and migration. The steady-state and transient-state density distribution of oxygen vacancies are formulated from the diffusion equation. The gas-sensing characteristics are simulated and following conclusions have been drawn:

(1) The performances of semiconductor gas sensors are found to be influenced by cooling rate during cooling or annealing process. A low cooling rate may enhance the sensor resistance and response to reducing gas. The annealing technique may recover the sensing ability of the quenched sample, the properties of which are raised from low values to the same level as the slowly-cooled one. It is inferred that a process that determines the sensor properties is interrupted by quenching and it is restarted by the subsequent annealing.
(2) The experimental phenomenon above leads to the investigation of oxygen vacancy behaviors during the fabrication process of semiconductor gas sensors. A diffusion equation is established based on the defect kinetics of diffusion and exclusion. The analytical solution illustrates the steady-state and transient-state distributions of oxygen vacancies in the grain. The behaviors of oxygen vacancies during sintering process are divided into four stages, which are namely formation, involvement, homogenization distribution and inhomogenization distribution.
(3) The gas-sensing characteristics of the semiconductor are simulated after the V_O_ density distribution expressions are incorporated with the Poisson’s equation in double Schottky model. The sensor resistance and response to reducing gas are both dependent on the time elapsed during the cooling process due to the migration of oxygen defects inside the grain. The validity of simulations is checked by the experimental results and are consistent with each other. The simulations infer that it is possible to control the sensor properties by interrupting the cooling process at a proper time in order to acquire a gas sensor with required characteristics. The GDOV model is also used to provide quantitative explanations for several key characteristics of semiconductor gas sensors, including the power law, grain size effect and effect of depletion layer width.
